# Supplementary material for: Pediatric anti-N-methyl-D-aspartate receptor (NMDAR) encephalitis: Exploring psychosis, related risk factors, and hospital outcomes in a nationwide inpatient sample: A cross-sectional study
Source: PLoS One. 2024 Feb 13;19(2):e0296870. doi: 10.1371/journal.pone.0296870 (PMC10863852; doi:10.1371/journal.pone.0296870)
Supplement: Attachment — Submitted filename: review.docx [file pone.0296870.s001.docx]

I am honored to have the opportunity to review **“**Title: Predictive Risk factors and Impact of Psychosis in Pediatric Encephalitis: A Cross-national Hospital Study**”.** This work includes a significant number of patients. However, I have a few comments and questions:

**Abstract:**

-In aim of the study, the authors aimed to evaluate the impact of hospital outcomes, while in the title they mentioned “Impact of Psychosis on Pediatric Encephalitis”.

-In line 53, it seems that a space is needed between patients. Encephalitis in patients with comorbid psychosis had a longer mean length of stay at 24.6 days.

-In conclusion, how identification of demographic predictors and comorbidities can aid in early recognition and intervention to optimize care and potentially reduce the healthcare burden?

**Methods:**

-Patients with encephalitis have been investigated for a year. It is not mentioned by which criteria psychosis was diagnosed. It is not clear which signs and symptoms are considered as psychosis. As the patients with encephalitis may have related symptoms such as hallucinations, delirium and other symptoms during the course of the disease.

-Hospital outcomes included hospital stay and charges. These are not impacts of psychosis on patients. On the other hand, due to the existence of more comorbidities in groups, how can they estimate the impact of psychosis alone?

**Results:**

-The data in text and tables are replicated. It is better to summarize the text and merge the tables.

-The etiology of encephalitis is not explained in this study, which is a major risk factor for encephalitis consequences.

**Discussion:**

-In line 192, Autoimmune encephalitis presentations associated with psychotic symptoms differ in terms of age and sex. When limbic encephalitis is prevalent in elderly (male or female? ) patients, then anti-NMDA-R encephalitis with psychotic symptoms is predominant in females, and children.
